# Supplementary material for: Hydrogen production in the presence of oxygen by Escherichia coli K-12
Source: Microbiology (Reading). 2022 Mar 28;168(3):001167. doi: 10.1099/mic.0.001167 (PMC9558352; doi:10.1099/mic.0.001167)
Supplement: Supplementary material 1 [file mic-168-1167-s001.pdf]

Supplementary Material

Hydrogen production in the presence of oxygen by *Escherichia coli* K-12

George D. Metcalfe [1], Frank Sargent [2] and Michael Hippler [1]*

[1] ***Department of Chemistry, University of Sheffield, Sheffield S3 7HF, UK***

[2] ***School of Natural & Environmental Sciences, Newcastle University, Newcastle upon Tyne NE1 7RU, UK***

* ***Corresponding author E-mail:* M.Hippler@sheffield.ac.uk**

Content:

S.1. Repeats of Fig. 1:
Anaerobic fermentation of glucose by E. coli MG1655

S.2. Repeats of Figs 3 and 4:
Anaerobic fermentation of glucose and exogenous formate (D- and ^{13}C-labelled in Figs 3 and 4, respectively) by E. coli MG1655

S.3. Repeats of Fig. 5:
Aerobic respiration of glucose by E. coli MG1655

S.4. Repeats of Fig. 6:
Aerobic respiration of glucose and exogenous formate-D by E. coli MG1655

S.5. Repeats of Fig. 7:
Aerobic respiration of glucose and exogenous formate-D by E. coli MG16dZ

S.1. Repeats of Fig. 1

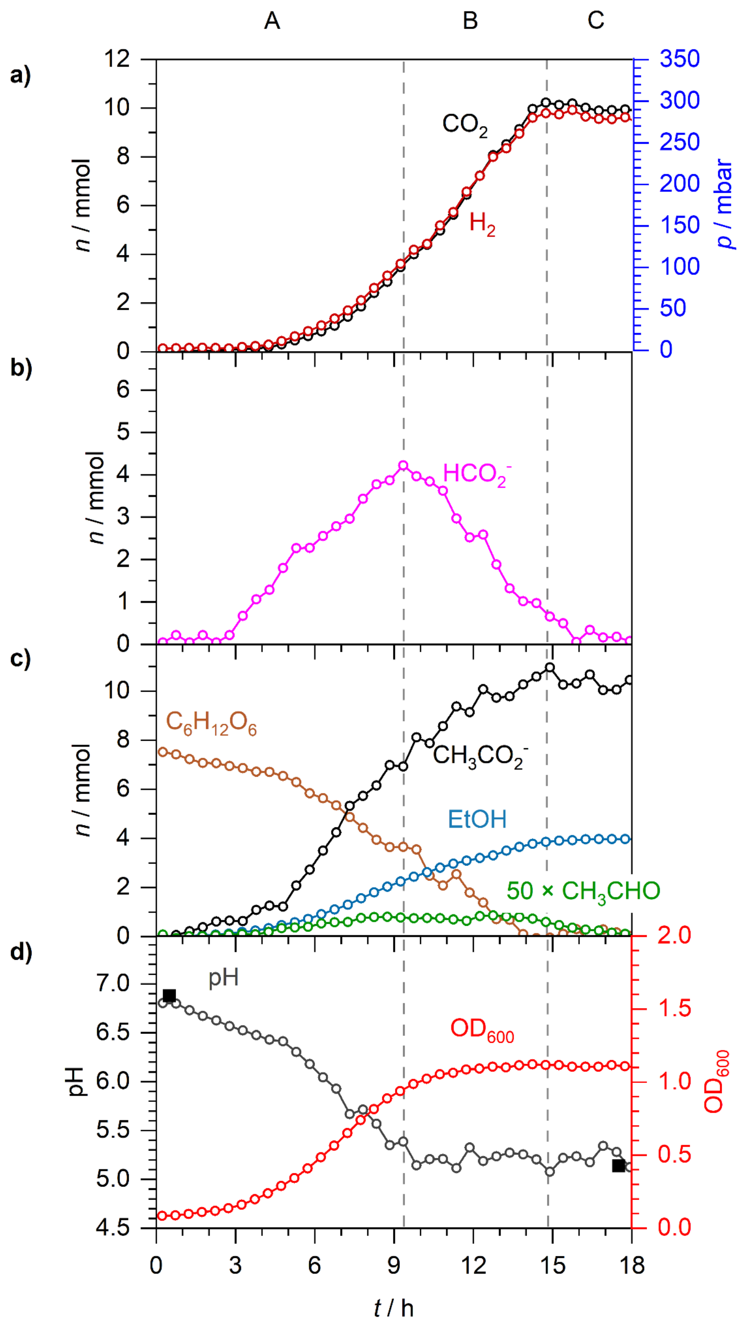

Fig. 1. Anaerobic fermentation of glucose by *E. coli* K-12.

Anaerobic fermentation by *E. coli* MG1655 during growth in M9 medium supplemented with 30 mM glucose. **A** to **C** denote three distinct phases: net formate excretion (A), net formate consumption (B) and formate depletion (C). **a)** Time-dependent number of moles (*n*) and equivalent partial pressures (*p*) of CO_2 and H_2. **b)** *n* of formate. **c)** *n* of acetaldehyde (×50), acetate, ethanol and glucose. **d)** Spectroscopically determined pH (open circles), externally measured pH (solid squares) and OD_{600}.

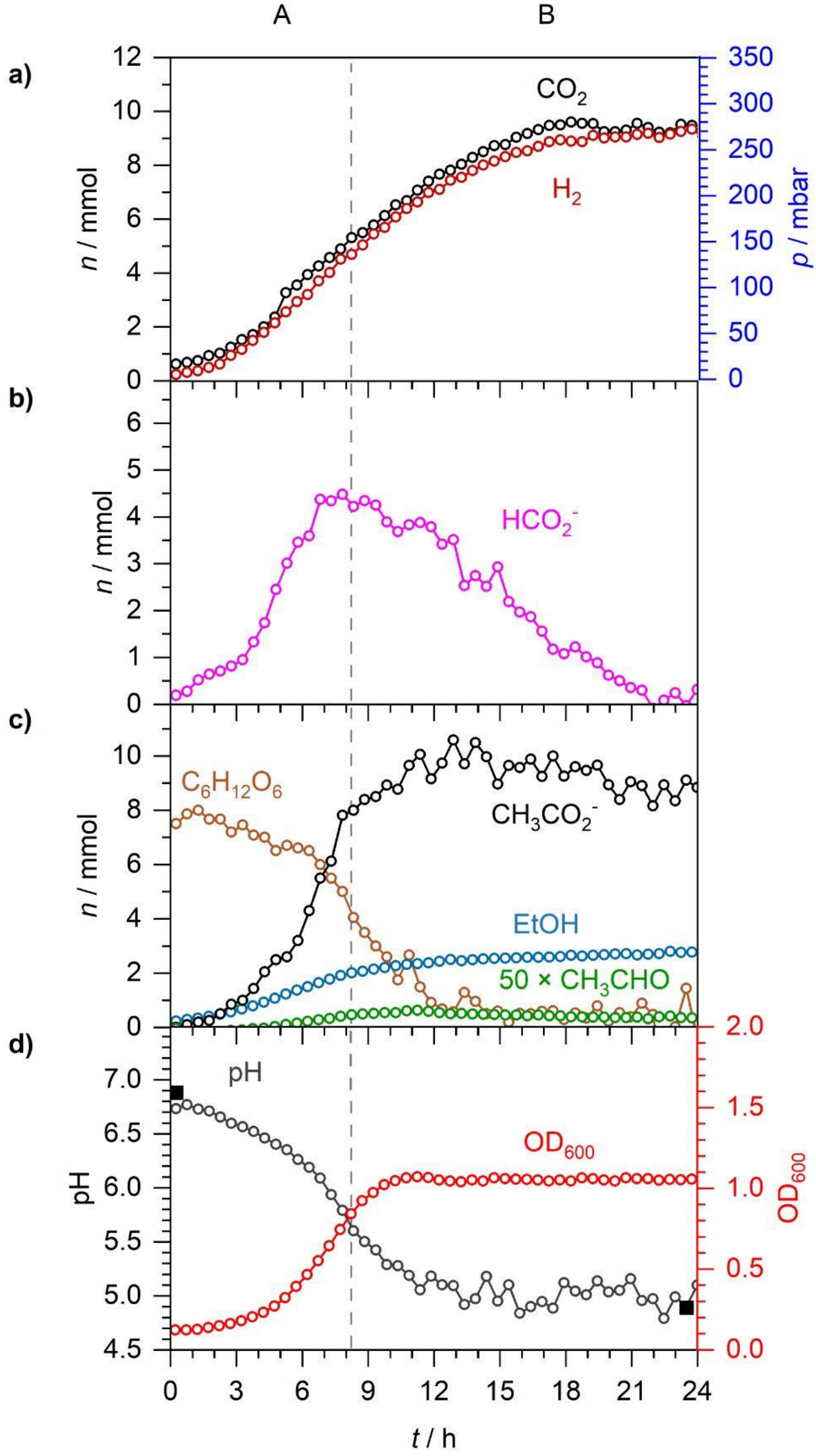

Fig. S1. A repeat of Fig. 1.

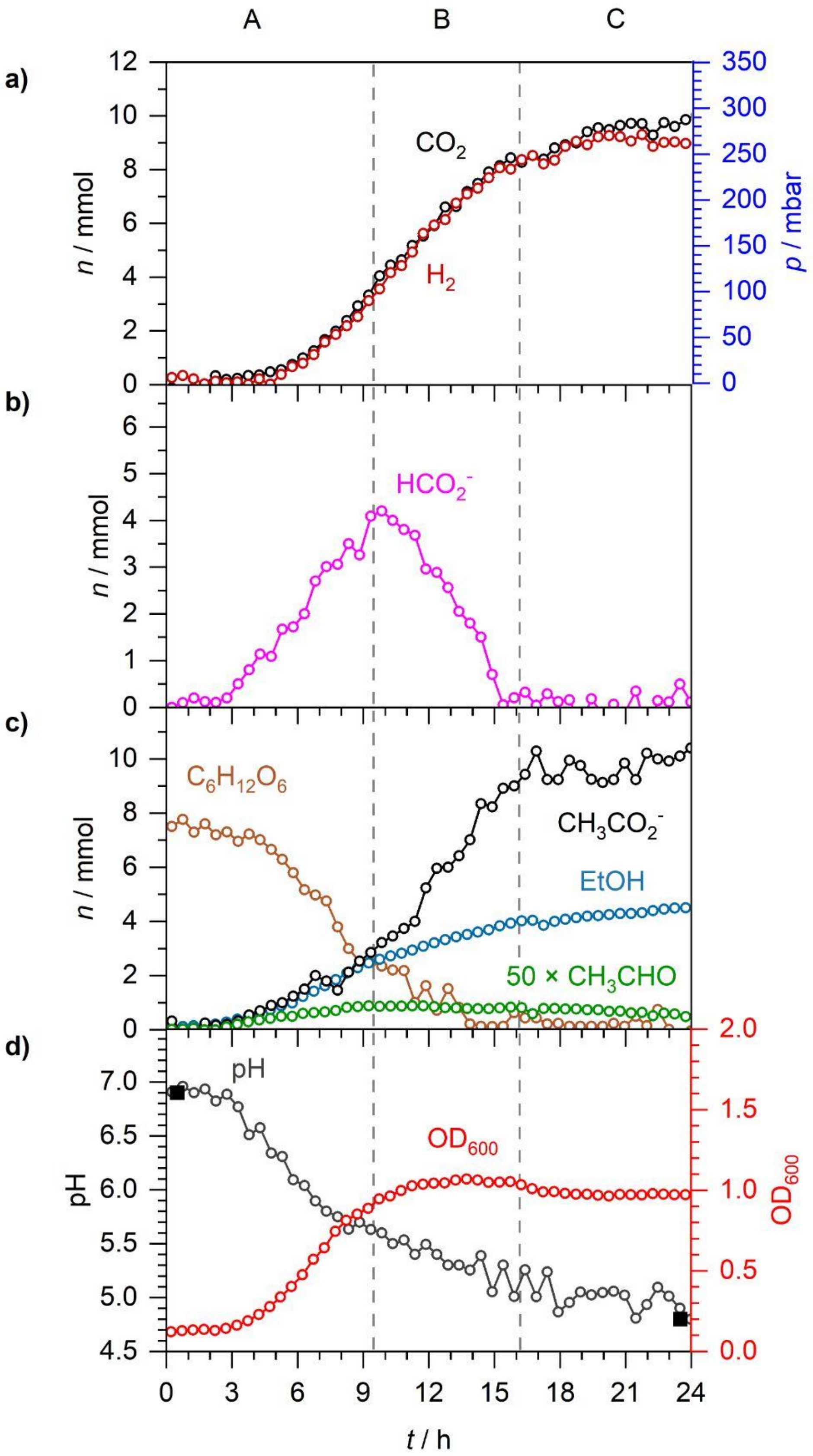

Fig. S2. A repeat of Fig. 1

S.2. Repeats of Figs 3 and 4

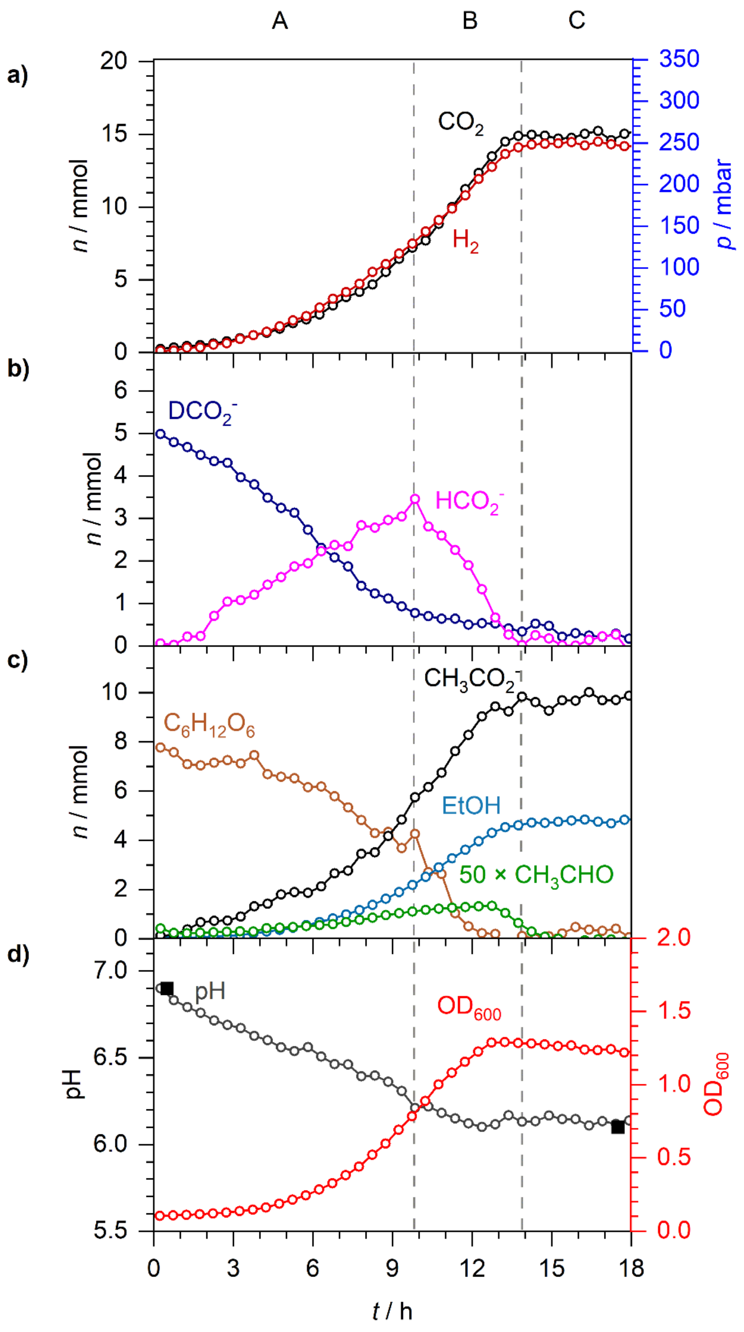

Fig. 3. Exogenous formate-D is taken up by the cell, increasing CO_2 and H_2 production.

Anaerobic fermentation by *E. coli* MG1655 during growth in M9 medium supplemented with 20 mM formate-D and 30 mM glucose. As before, **A** to **C** denote three distinct phases: net formate excretion (A), net formate consumption (B) and formate depletion (C). **a)** *n* and *p* of CO_2 and H_2. **b)** *n* of formate-D and formate. **c)** *n* of acetaldehyde (×50), acetate, ethanol and glucose. **d)** Spectroscopically determined pH (open circles), externally measured pH (solid squares) and OD_{600}.

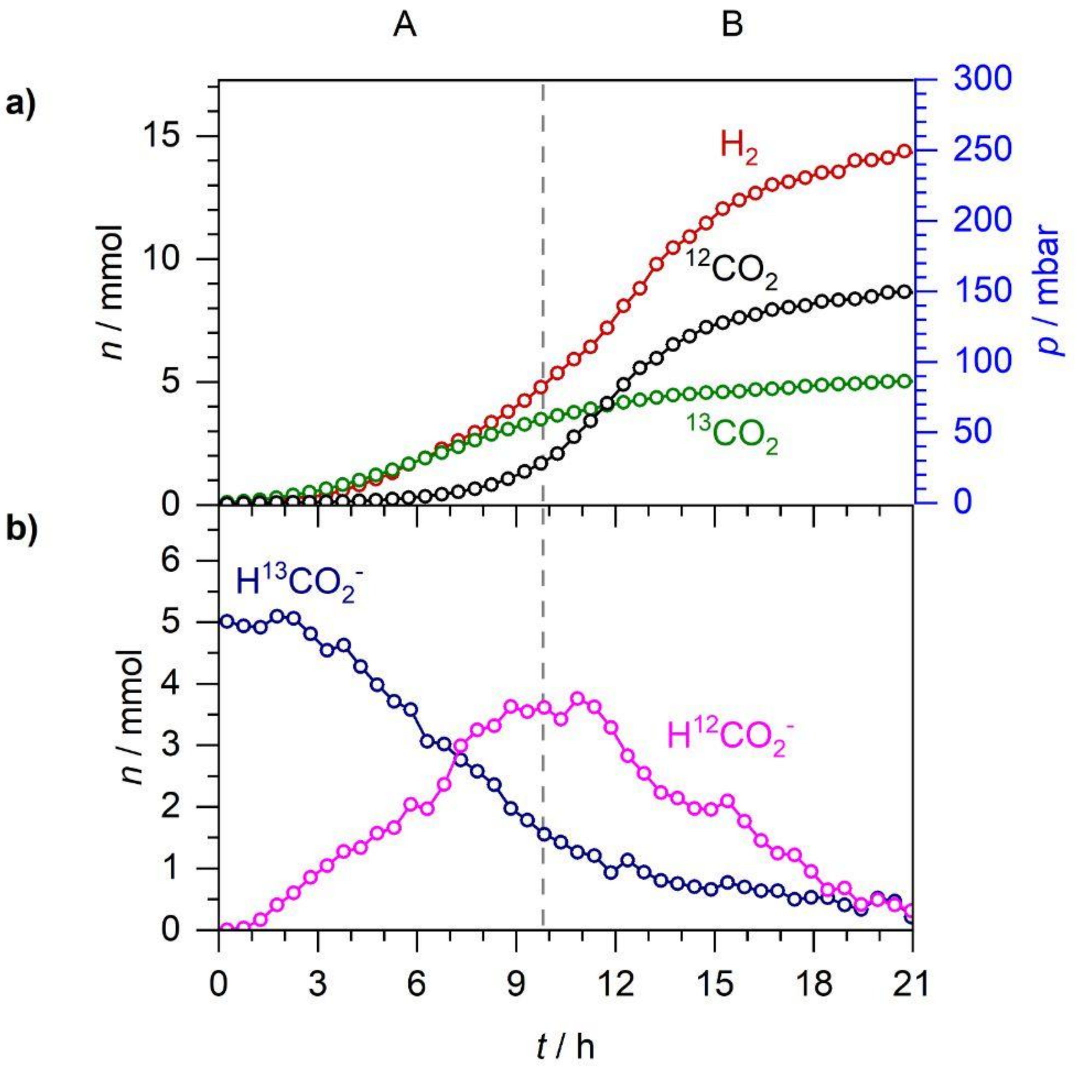

Fig. 4. CO_2 from exogenous and endogenous formate can be distinguished.

Anaerobic fermentation by *E. coli* MG1655 during growth in M9 medium supplemented with 20 mM formate-^{13}C and 30 mM glucose. **A** and **B** denote two distinct phases: net formate excretion (A) and net formate consumption (B). **a)** *n* and *p* of $^{13}CO_2$, $^{12}CO_2$ and H_2. **b)** *n* of formate-^{13}C and formate.

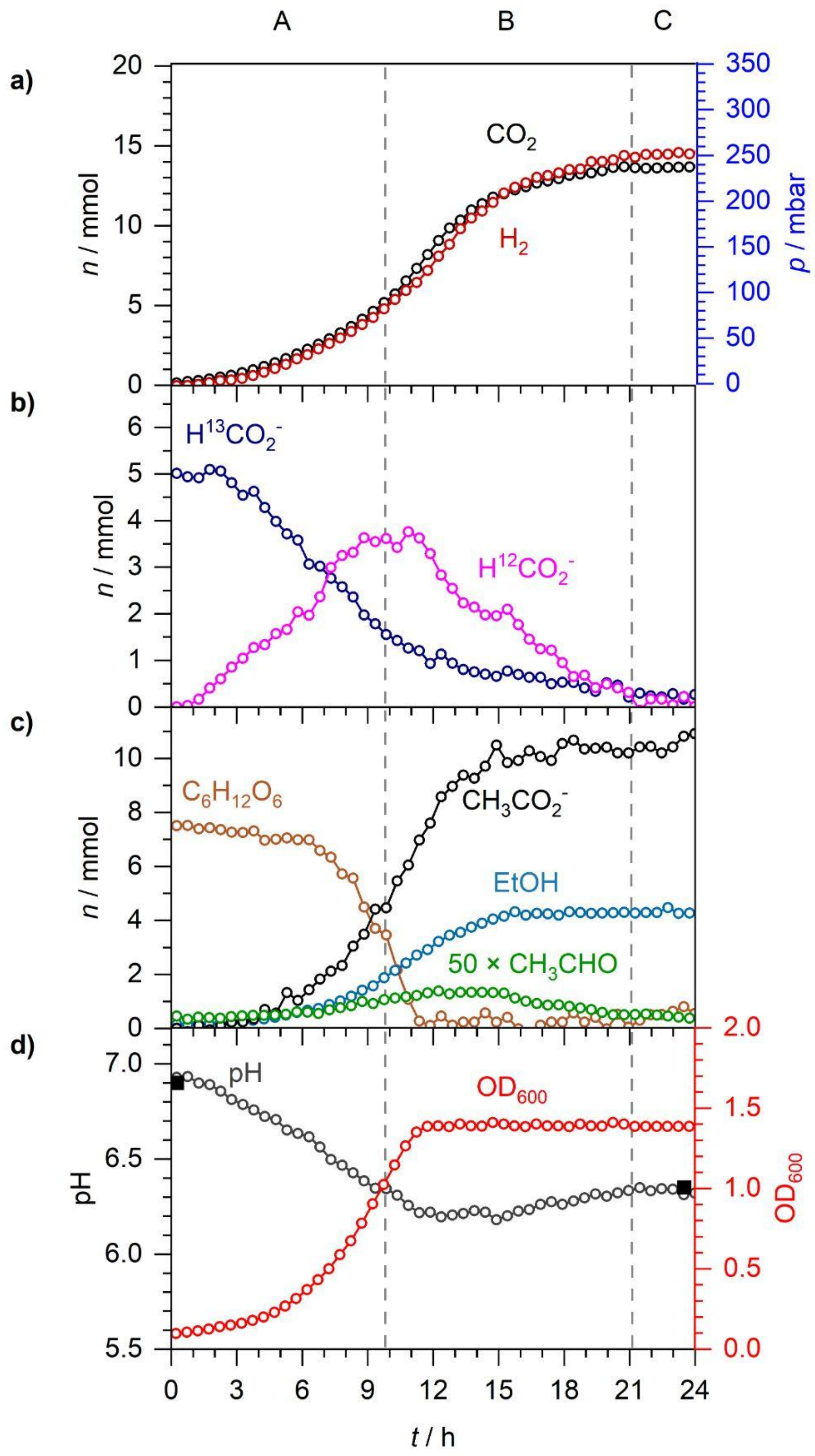

Fig. S3. The same experiment as in Fig. 4 but with all measured data displayed. Here, CO_2 is the sum total of $^{13}CO_2$ and $^{12}CO_2$. This also serves as a repeat of Fig 3.

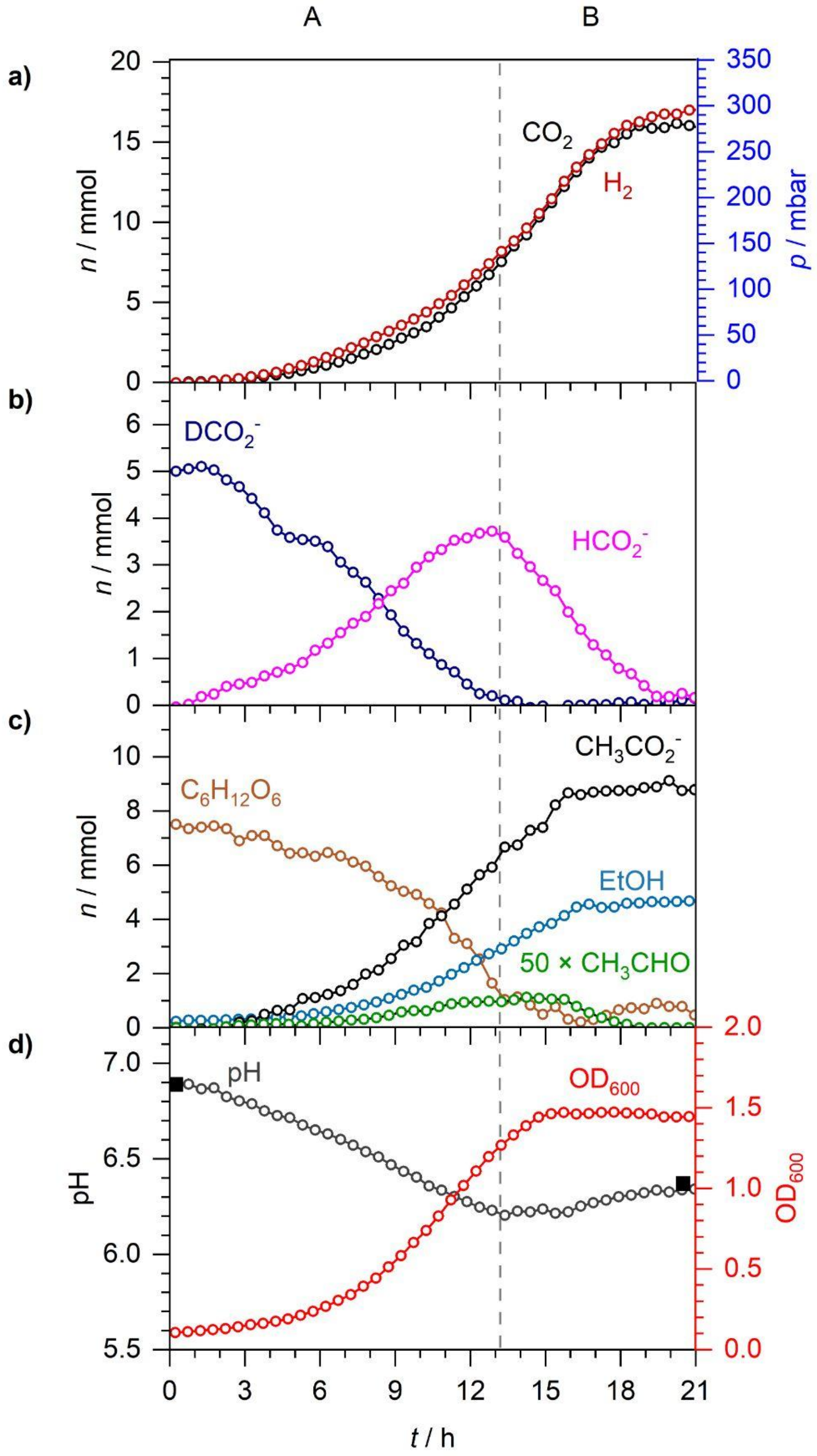

Fig. S4. A repeat of Figs 3 and 4.

S.3. Repeats of Fig. 5

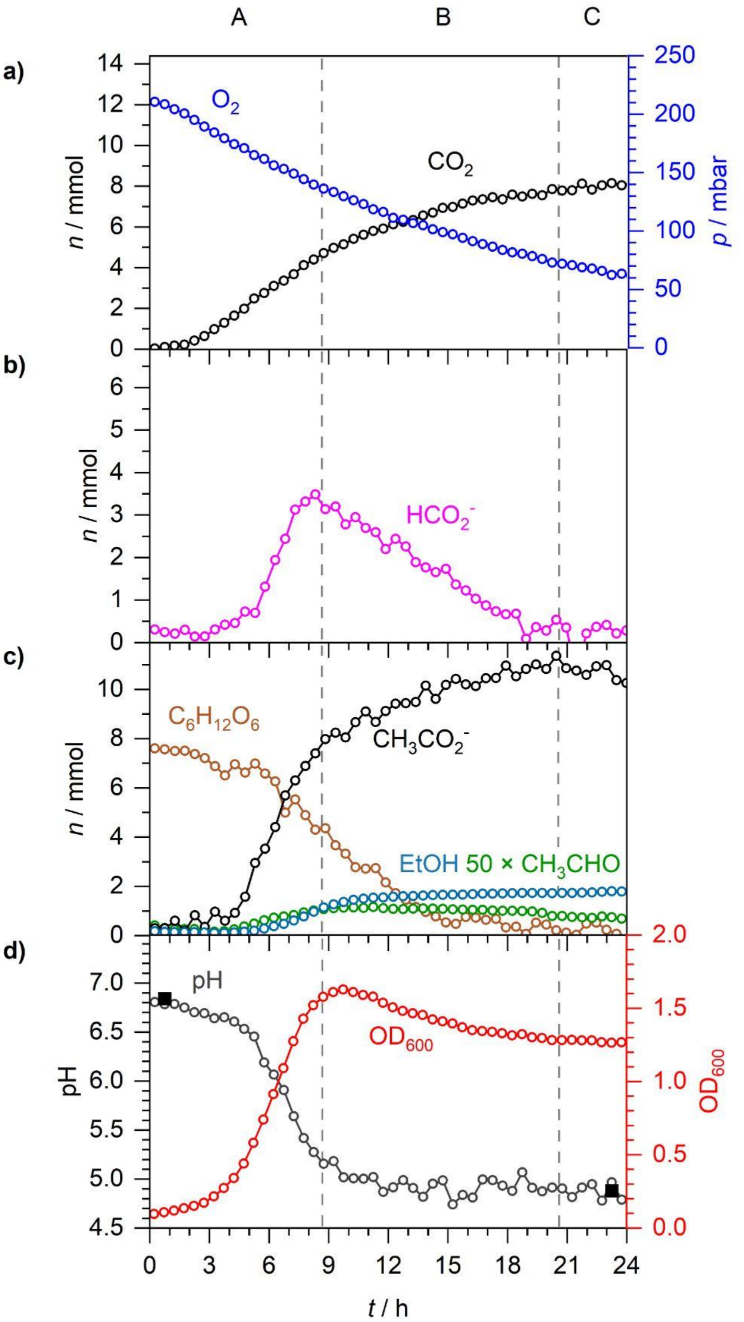

Fig. 5. Evidence for microaerobic metabolism combining aspects of aerobic respiration with anaerobic fermentation.

Aerobic respiration by *E. coli* MG1655 during growth in M9 medium supplemented with 30 mM glucose. **A** to **C** denote three distinct phases: net formate excretion (A), net formate consumption (B) and formate depletion (C). **a)** *n* and *p* of O_2 and CO_2. **b)** *n* of formate. **c)** *n* of acetaldehyde (×50), acetate, ethanol and glucose. **d)** Spectroscopically determined pH (open circles), externally measured pH (solid squares) and OD_{600}.

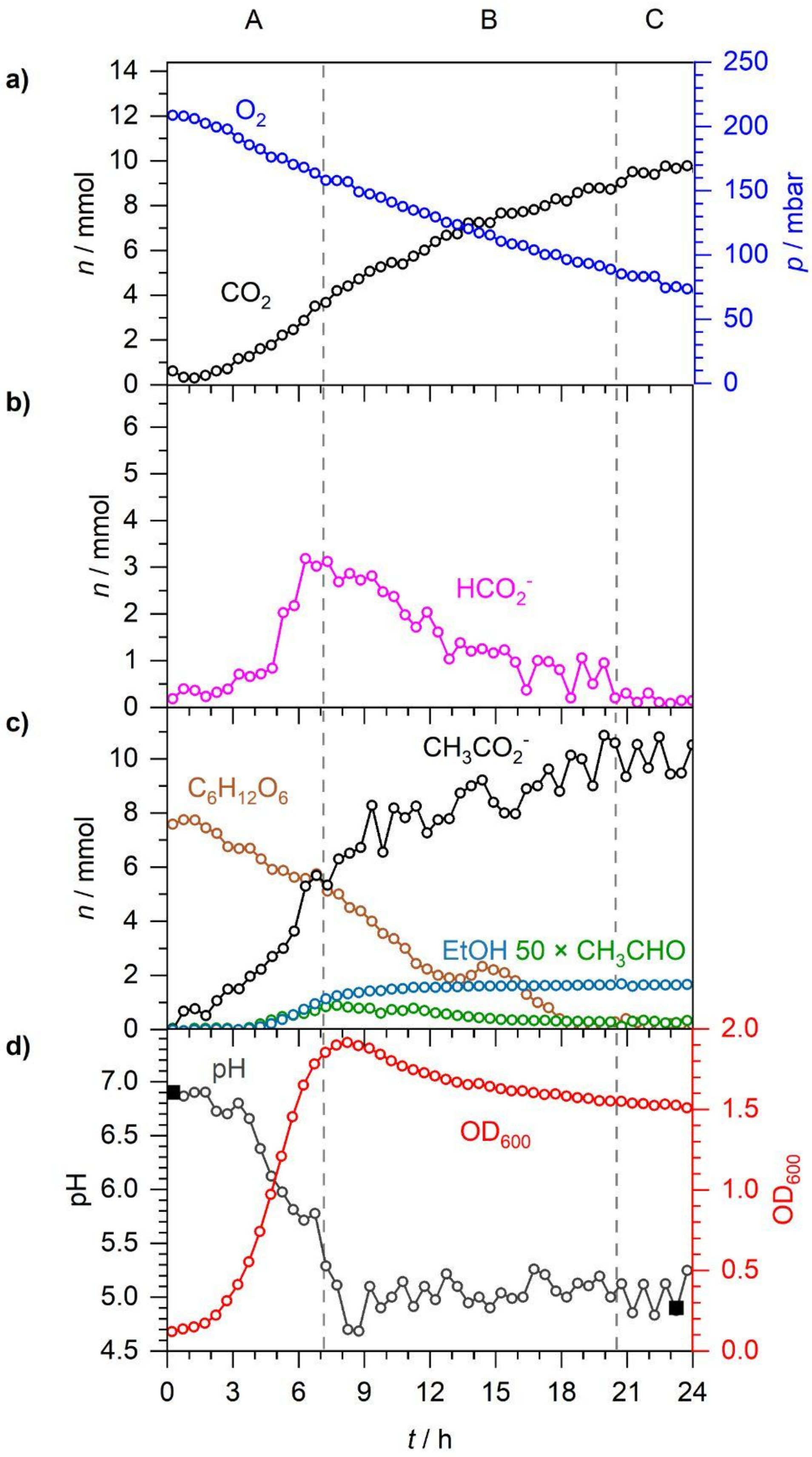

Fig. S5. A repeat of Fig. 5.

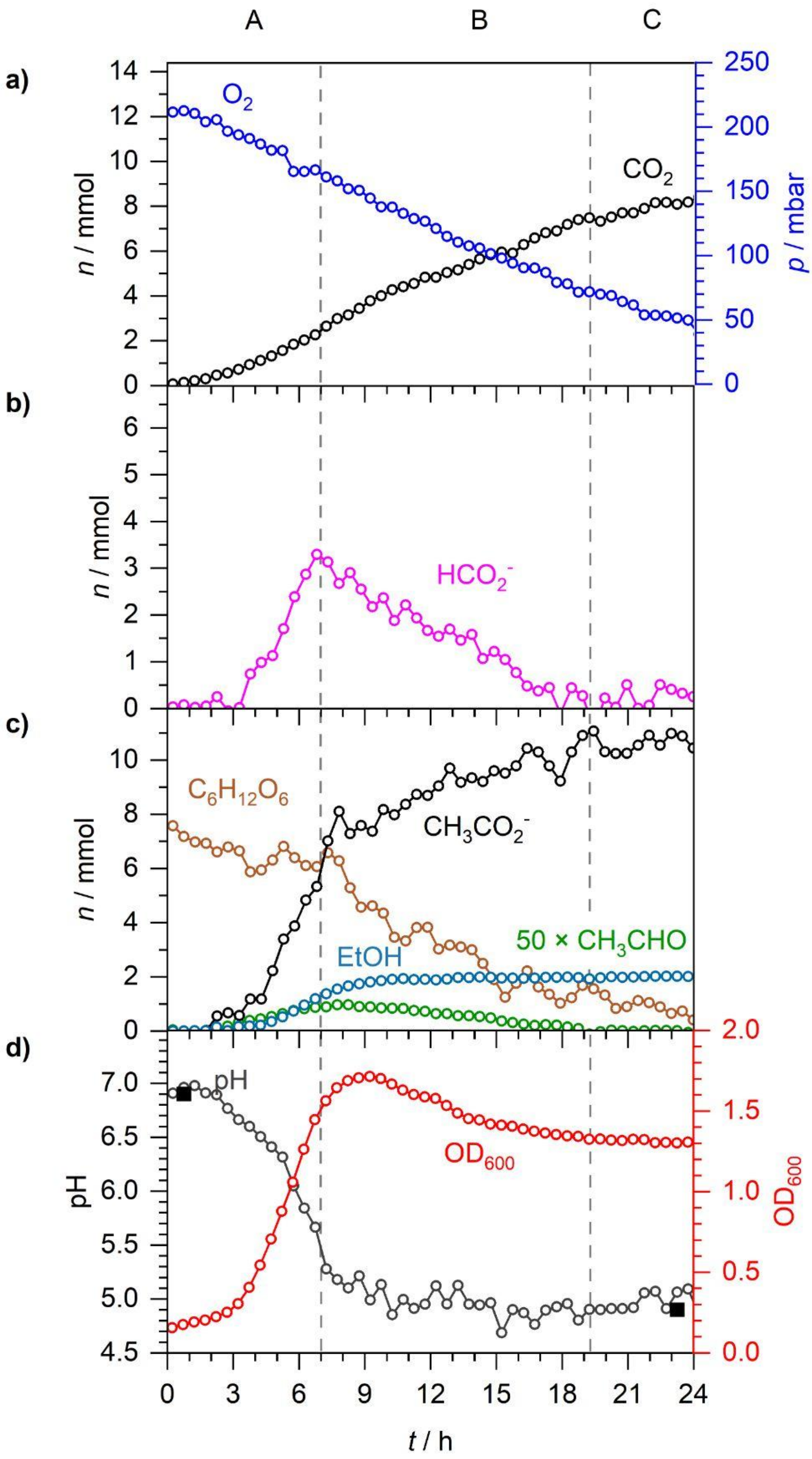

Fig. S6. A repeat of Fig. 5.

S.4. Repeats of Fig. 6

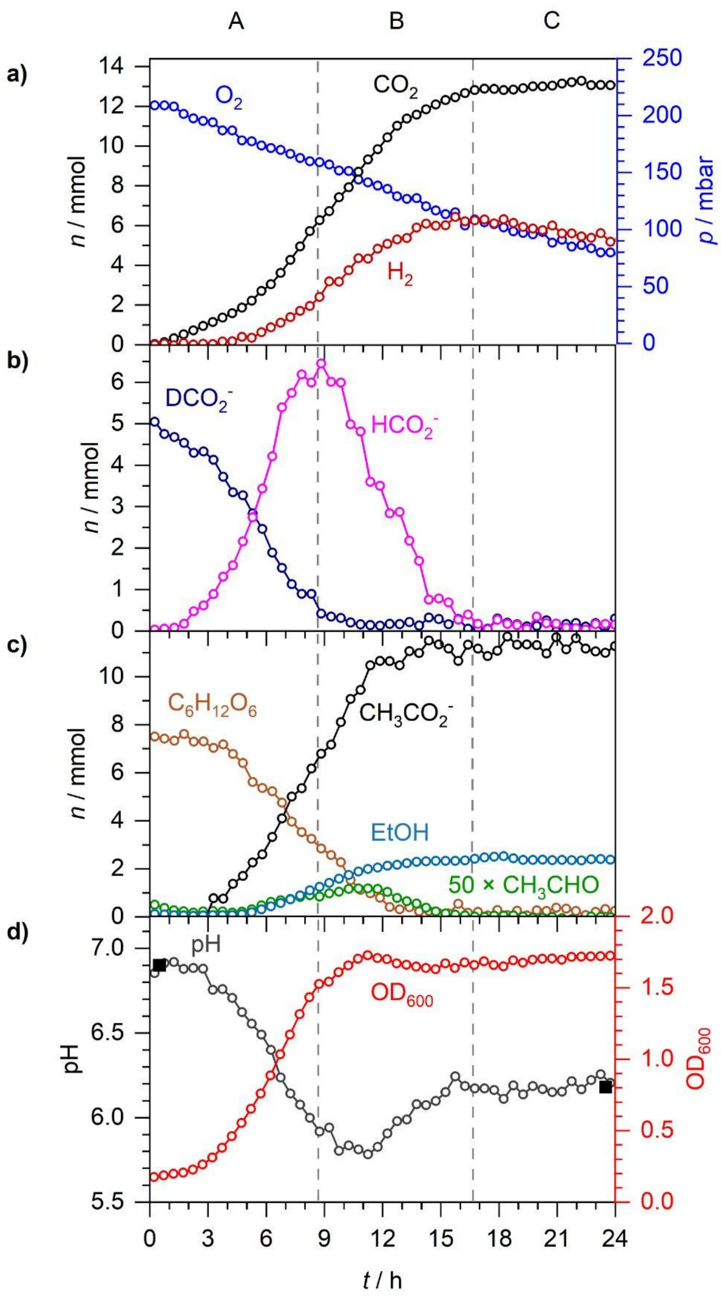

Fig. 6. Exogenous formate induces H_2 production during microaerobic respiration.

Aerobic respiration by *E. coli* MG1655 during growth in M9 medium supplemented with 20 mM formate-D 30 mM glucose. **A** to **C** denote three distinct phases: net formate excretion (A), net formate consumption (B) and formate depletion (C). **a)** *n* and *p* of O_2, CO_2 and H_2. **b)** *n* formate-D and formate. **c)** *n* of acetaldehyde (×50), acetate, ethanol and glucose. **d)** Spectroscopically determined pH (open circles), externally measured pH (solid squares) and OD_{600}.

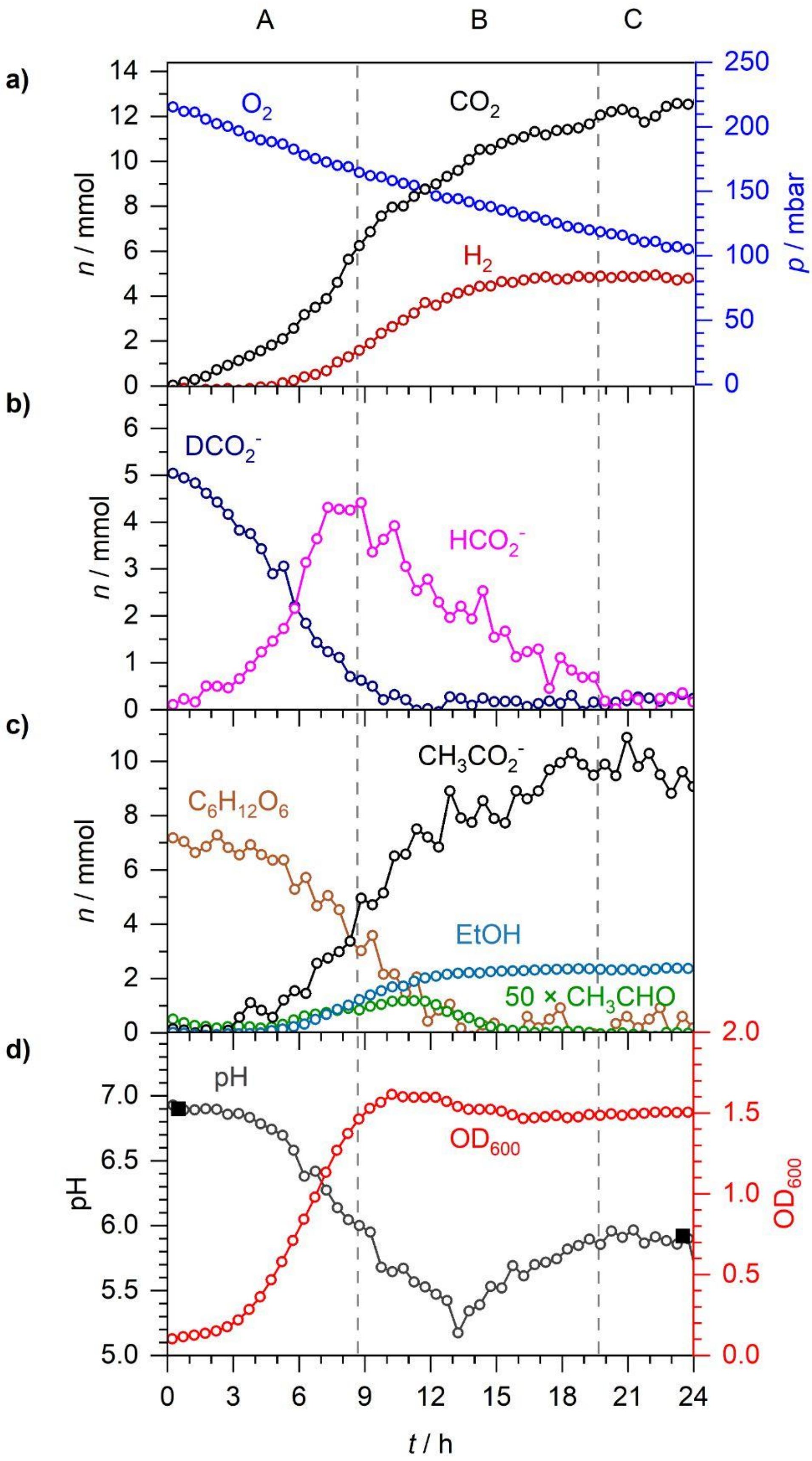

Fig. S7. A repeat of Fig. 6.

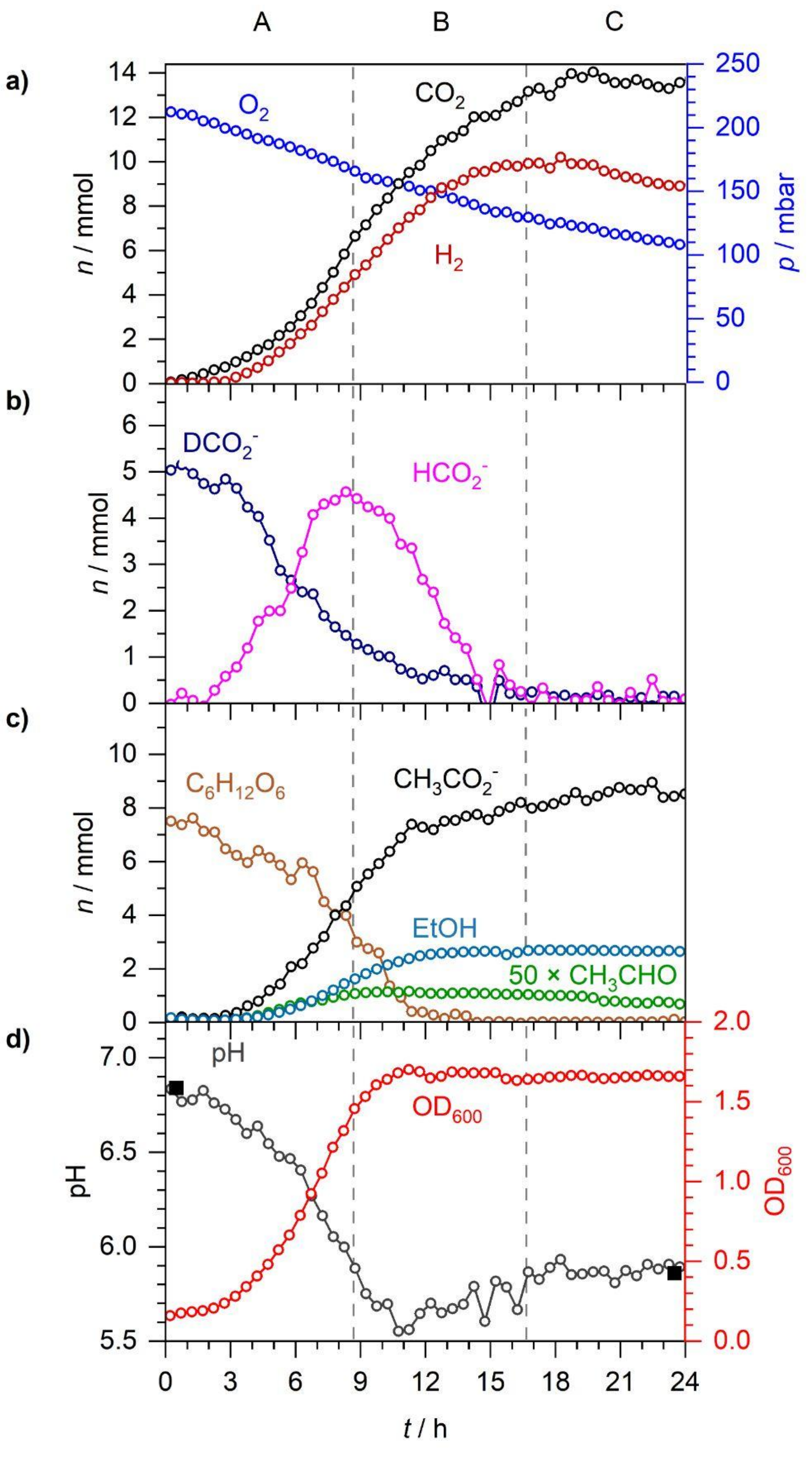

Fig. S8. A repeat of Fig. 6.

S.5. Repeats of Fig. 7

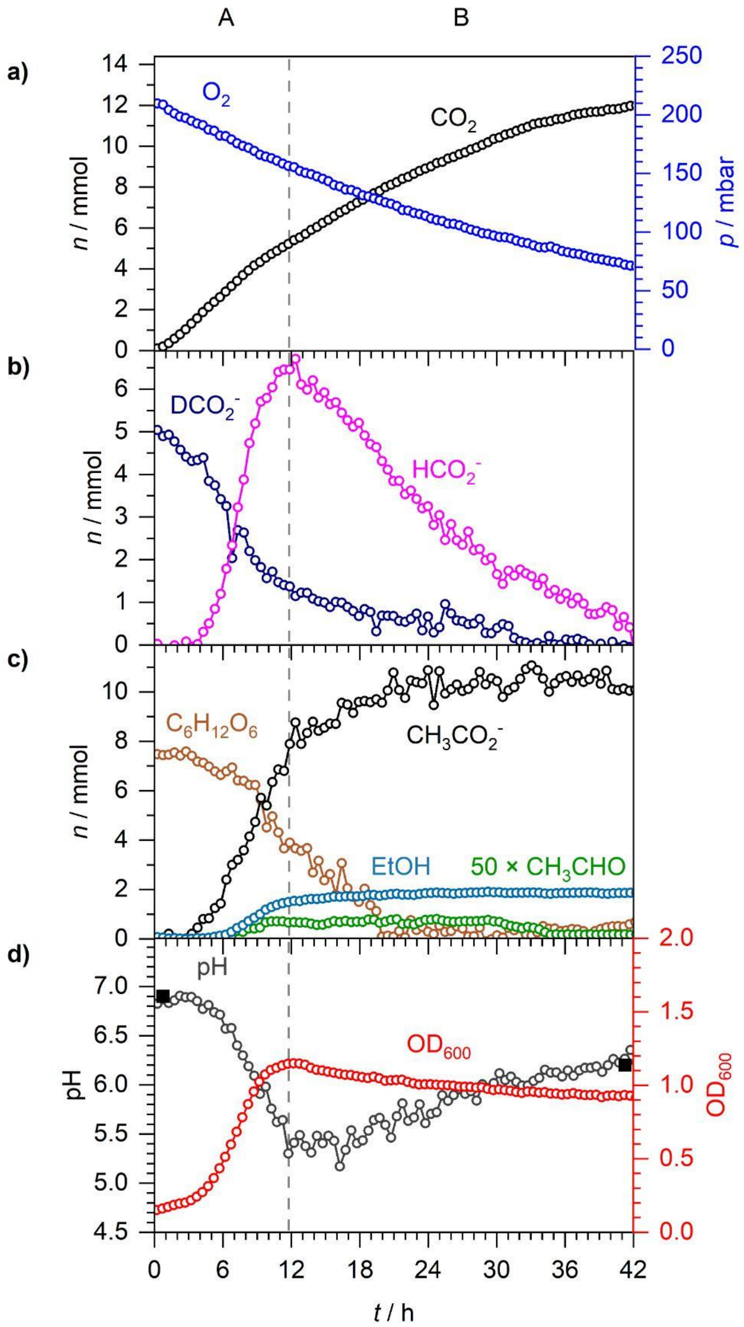

Fig. 7. H_2 production during microaerobic respiration is dependent upon FHL-1.

Aerobic respiration by *E. coli* MG16dZ (*ΔfdhF*) during growth in M9 medium supplemented with 20 mM formate-D 30 mM glucose. **A** and **B** denote three distinct phases: net formate excretion (A) and net formate consumption (B). **a)** *n* and *p* of O_2 and CO_2. **b)** *n* formate-D and formate. **c)** *n* of acetaldehyde (×50), acetate, ethanol and glucose. **d)** Spectroscopically determined pH (open circles), externally measured pH (solid squares) and OD_{600}.

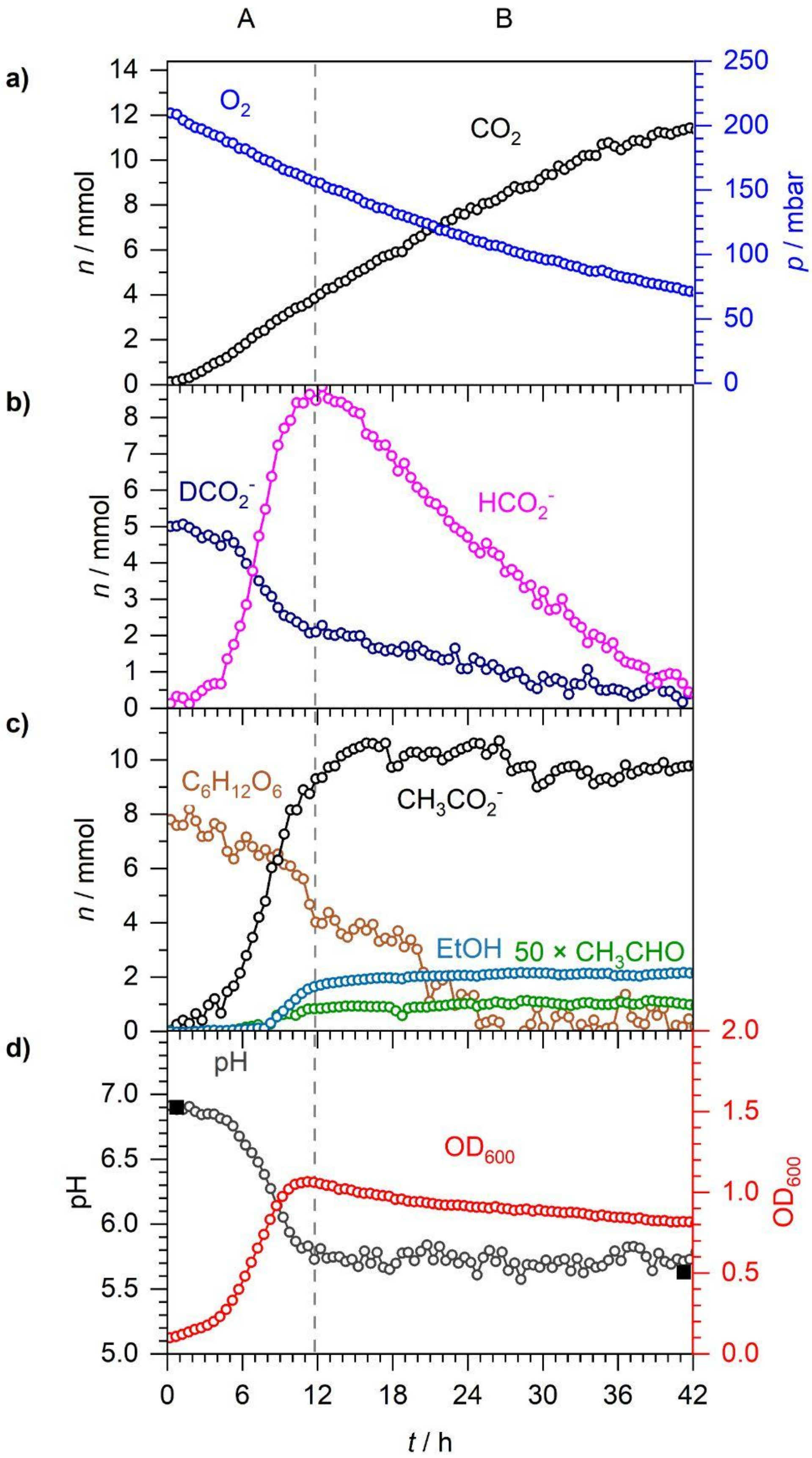

Fig. S9. A repeat of Fig. 7.

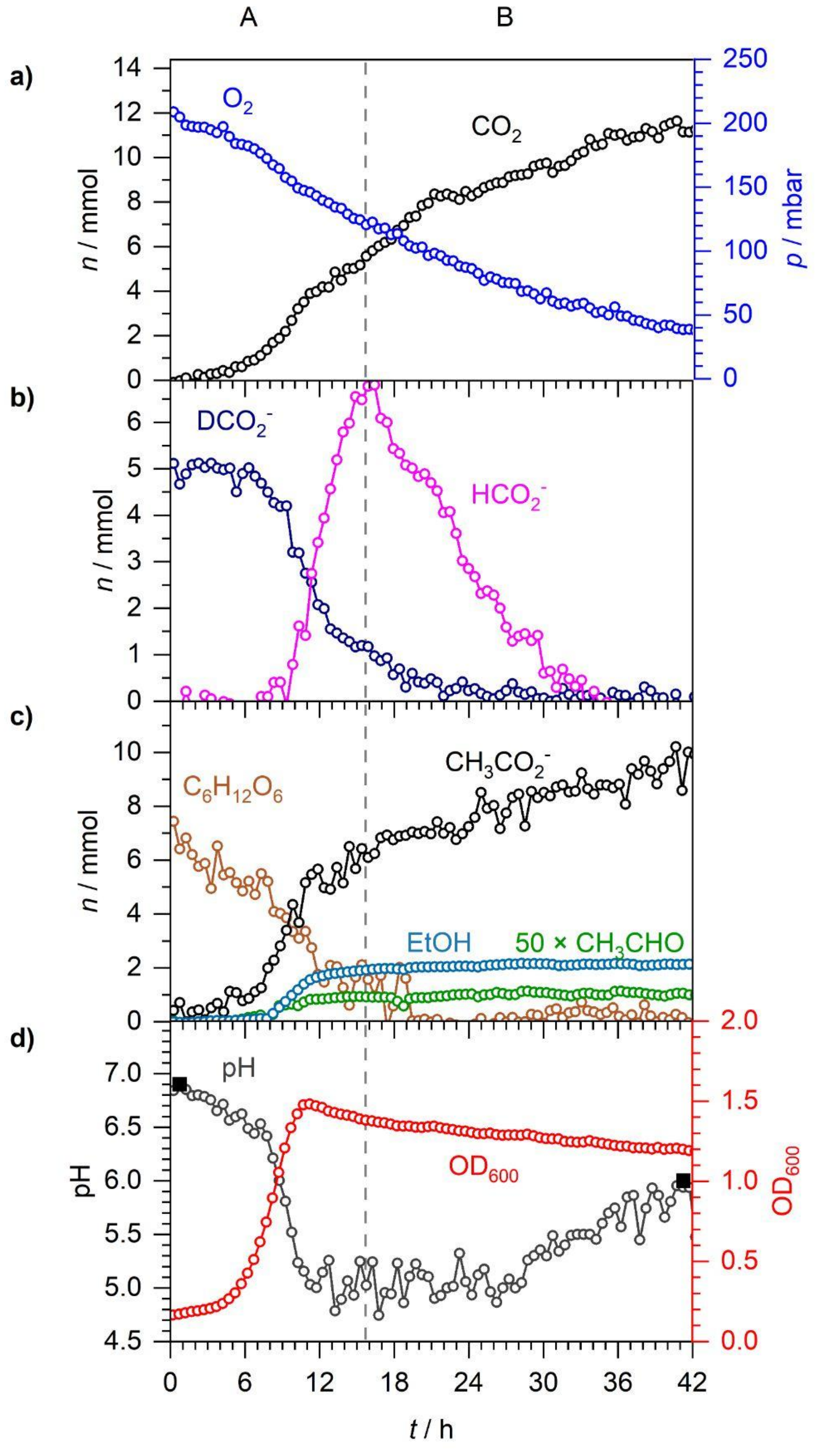

Fig. S10. A repeat of Fig. 7.
